# Promising treatment outcomes of intensity-modulated radiation therapy for nasopharyngeal carcinoma patients with N0 disease according to the seventh edition of the AJCC staging system

**DOI:** 10.1186/1471-2407-12-68

**Published:** 2012-02-15

**Authors:** Ying Sun, Ling-Long Tang, Lei Chen, Wen-Fei Li, Yan-Ping Mao, Li-Zhi Liu, Ai-Hua Lin, Li Li, Jun Ma

**Affiliations:** 1 State Key Laboratory of Oncology in South China, Department of Radiation Oncology, Sun Yat-sen University Cancer Center, No.651 Dongfeng Road East, Guangzhou 510060, People's Republic of China; 2 State Key Laboratory of Oncology in South China, Imaging Diagnosis and Interventional Center, Sun Yat-sen University Cancer Center, No.651 Dongfeng Road East, Guangzhou 510060, People's Republic of China; 3 Department of Medical Statistics and Epidemiology, School of Public Health, Sun Yat-sen University, No.74 Zhongshan Road, Guangzhou, People's Republic of China

## Abstract

**Background:**

Intensity-modulated radiation therapy (IMRT) provides excellent locoregional control for nasopharyngeal carcinoma (NPC), and has gradually replaced two-dimensional conventional radiotherapy as the first-line radiotherapy technique. Furthermore, in the new seventh edition of the American Joint Committee on Cancer (AJCC) staging system, retropharyngeal lymph nodes were upgraded from N0 to N1 disease as a result of their negative impact on the distant metastasis-free survival (DMFS) rates of NPC. This retrospective study was conducted in order to review the treatment outcomes and patterns of failure in NPC patients with N0 disease after IMRT in order to effectively guide treatment in the future.

**Methods:**

We retrospectively reviewed data from 506 biopsy-proven nonmetastatic NPC patients. There were 191 patients with negative cervical lymph node involvement. According to the seventh edition of the American Joint Committee on Cancer (AJCC) staging system, 110 patients (21.7%) were staged with N0 disease, and 81 patients (16.0%) were reclassified with N1 disease due to the presence of RLN metastasis. All patients received IMRT as the primary treatment.

**Results:**

In patients with negative cervical lymph node involvement, distant metastasis-free survival (DMFS) was significantly higher in patients without retropharyngeal lymph node (RLN) metastasis than those with RLN metastasis (95.9% vs. 88.1% respectively, P = 0.04). For N0 disease, the 5-year overall survival (OS), local relapse-free survival (LRFS), nodal relapse-free survival (NRFS) and DMFS rates were 93.8%, 97.1%, 99.1% and 95.9%, respectively. For T1N0, T2N0, T3N0 and T4N0, OS was 97.8%, 100%, 93.8% and 76.9%, LRFS was 100%, 92.9%, 100% and 88.9% and DMFS was 96.6%, 90.9%, 100% and 93.3%, respectively. OS and LRFS were higher in T1-3 N0 patients than T4N0 patients (*P *< 0.01 and *P *= 0.01, respectively).

**Conclusions:**

The seventh edition of the AJCC N-staging system improves prognostic accuracy by upgrading RLN metastasis to N1 disease. IMRT produces excellent survival rates in T1-3 N0 disease; however, T4N0 disease remains a challenge and additional improvements are required to achieve a favorable prognosis for these NPC patients.

## Background

Although nasopharyngeal carcinoma (NPC) is rare in most of the world, it is endemic in certain regions, especially Southeast Asia. The incidence of NPC in Southern China is approximately 30 to 80 per 100,000 people per year [1]. Newly diagnosed NPC without distant metastasis is typically treated with radiotherapy, rather than by surgical intervention [2].

The tumor-node-metastasis (TNM) staging system for NPC is used to evaluate prognosis, aid treatment planning and facilitate the stratification of treatment. In general, the T-classification is prognostic of local control, whereas the N-classification can significantly predict neck and distant control [3]. In N0 disease, the T-classification is the major factor which affects prognosis. When N0 patients are treated with two-dimensional conventional radiotherapy, the 5-year overall survival (OS) rate is 73%-87%, and the local relapse-free survival (LRFS) rate is 88.6% [4-6]. Concurrent chemotherapy/radiotherapy plus adjuvant chemotherapy is recommended for T2-4 N0 disease by the National Comprehensive Cancer Network (NCCN) clinical practice guidelines.

Recently, intensity-modulated radiotherapy (IMRT) has gradually replaced two-dimensional conventional radiotherapy as the primary radiotherapy technique. Compared to two-dimensional conventional radiotherapy, IMRT improves tumor target conformity and allows safer dose escalations [7]. Lee et al. reported that the 4-year local progression-free survival rate after IMRT was 97% [8], and subsequent studies have achieved similar results [9,10]. As the locoregional control rate achieved using IMRT is greater than 90%, the prognostic power of the T-classifications has decreased [11], and excellent results can potentially be obtained using IMRT in N0 disease.

The TNM system of the seventh edition of the American Joint Committee on Cancer (AJCC) staging system for NPC considers metastasis of the retropharyngeal lymph nodes (RLN). The classification of negative cervical lymph nodes with RLN metastasis as N0 disease in the sixth edition of the AJCC staging system has been upgraded to N1 disease in the seventh edition [12]. Tang et al. [13] and Tham et al. [14] reported that patients with RLN metastasis and negative cervical lymph nodes have a similar risk of distant metastasis to N1 disease, and proposed that patients with RLN metastasis should be classified with N1 disease, regardless of laterality. Therefore, patients classified with N0 disease according to the seventh edition of the AJCC staging system, where RLN metastasis has been upgraded to N1 disease, may have a better prognosis than patients staged with N0 disease according to the previous editions.

Only few studies have reported the treatment outcomes after IMRT in NPC patients with N0 disease according to the seventh edition of the AJCC staging system, and additional data on the recurrence patterns and survival rates is required to generate new treatment strategies. This study was conducted in order to determine the treatment outcomes and the patterns of failure in NPC patients with N0 disease after IMRT, in order to more effectively guide future treatment.

## Methods

### Patient characteristics

This retrospective study was approved by the Institutional Review Board of Sun Yat-Sen University, Guangzhou, China. All NPC patients treated with IMRT at the Sun Yat-Sen University Cancer Center between January 2003 and December 2006 were eligible, and 506 patients with newly diagnosed, nonmetastatic, histologically proven NPC disease were enrolled in the study.

All patients completed a pre-treatment evaluation which included a complete patient history, physical examination, hematology and biochemistry profiles, MRI scan of the neck and nasopharynx, chest radiography, abdominal sonography and a whole body bone scan using single photon emission computed tomography. In addition, 130 (25.4%) patients also underwent a positron emission tomography-computed tomography (PET-CT) scan. All patients were restaged according to the seventh edition of the AJCC system [12]. The distribution of T classification, N classification, and overall stage was as follows: T1: 162 patients (32.0%); T2: 77 patients (15.2%); T3: 127 patients (25.1%); T4: 140 patients (27.7%);

N0:110 patients (21.7%); N1: 252 patients (49.8%); N2: 97 patients (19.2%); N3: 47 patients (9.3%); stage I: 60 patients (11.9%); stage II: 118 patients (23.3%); stage III: 150 patients (29.6%); and stage IV: 178 patients (35.2%). A total of 81 patients that were negative for spread to cervical lymph nodes (16.0%) were reclassified with N1 disease due to the presence of RLN metastasis.

Of the 110 patients with N0 disease, the median age of the cohort was 43 years (range: 13-75 years), and included 92 males and 18 females (male/female ratio, 5.1:1). Histologically, 0.9% of cases were classified as World Health Organization (WHO) grade I disease and 99.1% were WHO grade II/III disease (Table 1).

**Table 1:** Clinicopathological features in the study population of 110 nasopharyngeal carcinoma patients with N0 disease, according to the seventh edition of the American Joint Committee on Cancer (AJCC) staging system


**Characteristics**	**Number of patients (%)**

*Age (years)*	
< 50	76 (69.1%)
≥50	34(30.9%)
*Gender*	
Male	92 (83.6%)
Female	18 (16.4%)
*Pathological features*	
WHO grade I	1 (0.9%)
WHO grade II/III	109 (99.1%)
*T-category*	
T1	60 (54.5%)
T2	16 (14.5%)
T3	16 (14.5%)
T4	18(16.4%)

### Imaging protocol

All patients underwent MRI scans using a 1.5-T system (Signa CV/i; General Electric Healthcare, Chalfont St. Giles, United Kingdom) and the region from the suprasellar cistern to the inferior margin at the sternal end of the clavicle was examined with a head-and-neck combined coil. T1-weighted fast spin-echo images in the axial, coronal, and sagittal planes (repetition time, 500-600 ms; echo time, 10-20 ms), and T2-weighted fast spin-echo MR images in the axial plane (repetition time, 4000-6000 ms; echo time, 95-110 ms) were obtained before injecting the contrast material. After intravenous administration of gadopentetate dimeglumine (Gd-DTPA; Magnevist, Schering, Berlin, Germany) at a dose of 0.1 mmol/kg, spin-echo T1-weighted axial and sagittal sequences and spin-echo T1-weighted fat-suppressed coronal sequences were performed sequentially, with the same parameters used prior to Gd-DTPA injection, using a section thickness of 5 mm and a matrix size of 512 × 512. All MR images were reviewed by two radiologists with more than 10 years of experience in the MR imaging of head and neck cancers. All images were evaluated independently, and disagreements were resolved by consensus.

### Treatment

All patients were treated by IMRT using the PEACOCK-MIMiC system (Corvus^® ^3.0; Nomos Corporation, Alderley Qld, Australia) and VARIAN 6MV-photons [15]. In brief, patients were immobilized in a supine position using a thermoplastic mask. After administration of intravenous contrast material, 3 mm CT slices depicting the head area were acquired until 2 cm below the sternoclavicular joint. The primary tumor area and the upper-neck area above the caudal edge of the cricoid cartilage were treated by IMRT. Target volumes were delineated according to our institutional treatment protocol, in agreement with the International Commission on Radiation Units and Measurements Reports 50 and 62. The clinical target volumes (CTV) were individually delineated on the basis of the tumor invasion pattern. The prescribed radiation dose was applied as follows: a total dose of 68 Gy in 30 fractions at 2.27 Gy/fraction to the planning target volume (PTV) of the gross tumor volume of the primary tumor (GTV-P), 60-64 Gy to the PTV of the nodal gross tumor volume (GTV-N), 60 Gy to the PTV of CTV-1 (i.e., the high-risk regions), and 54 Gy to the PTV of CTV-2 (i.e., the low-risk regions) and CTV-N (i.e., the neck nodal regions). A total of 43.6% (48/110) of the N0 disease patients only received prophylactic irradiation to the upper neck lymph drainage region, including Levels II, III and VA of the upper neck lymph nodes, excluding the Level IV and VB and supraclavicular lower neck lymph node drainage areas. For the remainder of the N0 and all N1-3 disease patients, an anterior cervical field was used for irradiation of the lower neck. All patients were treated with one fraction daily for 5 days per week.

Chemotherapy was administered to 88.6% (295/333) of the patients with Stage III or IV disease and 82.3% (28/34) of the patients with T3-4 N0 disease. Chemotherapy included concurrent chemotherapy alone, concurrent chemotherapy combined with induction chemotherapy and/or adjuvant chemotherapy in conjunction with a platinum-based therapeutic clinical trial. Details of the chemotherapy agents and dose intensity used are provided in Table 2. When possible, salvage treatments, including afterloading, surgery and chemotherapy were provided in the event of documented relapse or persistent disease.

**Table 2:** Details of the chemotherapy schedule and doses used for stage T3-4 N0 nasopharyngeal carcinoma patients.


**Mode of treatment**	**Number of patients**	**%**

RT alone	6	17.6
CCRT	7	20.5
NAC + RT	2	5.9
NAC + CCRT	16	47.1
NAC + CCRT + ADC	3	8.8

CCRT, concomitant chemo-radiotherapy (cisplatin, 80 mg/m^2 ^on day 1, 22 and 43 or 40 mg/m^2 ^weekly); NAC, neoadjuvant chemotherapy (cisplatin, 80 mg/m^2 ^day 1; 5-flourouracil, 800 mg/m^2 ^days 1-5); RT, radiotherapy; ADC, adjuvant chemotherapy (cisplatin, 80 mg/m^2 ^day 1; 5-flourouracil, 800 mg/m^2 ^days 1-5).

### Follow-up

After the completion of radiotherapy, all patients were followed up every 1-3 months during the first 2 years, every 6 months in years 2 to 5 and annually thereafter. Each follow up included a physical examination, flexible endoscopy, basic serum chemistry, chest x-ray, liver and abdomen ultrasound and bone scan. MRI of the head and neck was performed approximately 3 months after the completion of IMRT and every 6-12 months thereafter. The median follow-up period was 56.8 months (range: 3.1-102 months).

### Statistical methods

The following end points were assessed: overall survival (OS), disease-specific survival (DSS), local relapse-free survival (LRFS), nodal relapse-free survival (NRFS) and distant metastasis-free survival (DMFS). Time was measured from the start of treatment (radiotherapy and/or chemotherapy) to the time of the first failure or last examination. OS was measured from the date of first radiotherapy or chemotherapy to death due to any cause. Patient who were alive were censored. DSS was defined as time to death as a result of NPC, or as a result of toxicity from radiotherapy or chemotherapy. As for DSS, deaths that were unrelated to NPC or treatment were censored. Similar definitions were used for LRFS and DMFS analyses. SPSS 11.0 software was used for the statistical analysis. The actuarial rates were calculated using the Kaplan-Meier method [16], and the differences were compared using the log-rank test. Multivariate analyses with the Cox proportional hazards model were used to test the independent significance of the predictors [17], and two-tailed *P *values less than 0.05 were considered statistically significant.

## Results

### Treatment outcomes

The 5-year survival rates were as follows: OS, 83.4%; LRFS, 93.7%; NRFS, 97.2%; and DMFS, 84.8%. A total of 38 patients (7.5%) developed locoregional recurrence, 74 patients (14.6%) developed distant metastases, and 81 patients (16.0%) died. In addition, 11 patients (2.2%) developed both locoregional recurrences and distant metastases, 18 patients (3.6%) had isolated local recurrences, 7 patients (1.4%) had isolated regional recurrences and 63 patients (12.5%) had isolated distant metastases.

**Figure 1:**
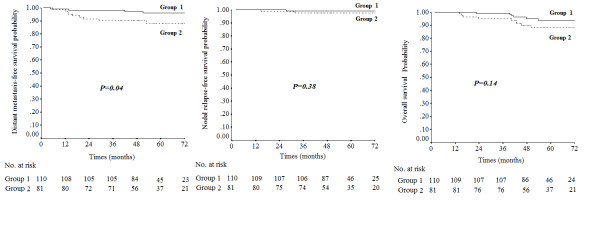
**Distant metastasis-free survival, nodal relapse-free survival and overall survival of nasopharyngeal carcinoma patients with negative cervical lymph node involvement stratified according to the absence of retropharyngeal lymph node (RLN) metastasis (Group 1) and presence of RLN metastasis (Group 2)**.

The 191 patients with negative cervical lymph node involvement were divided 2 groups. Group 1 included the 110 patients without RLN metastasis, and Group 2 included the 81 patients with RLN metastasis. DMFS was significantly higher in Group 1 than Group 2 (5-year DMFS: 95.9% vs. 88.1%, P = 0.04). No significant difference was observed in the NRFS and OS of Group 1 and Group 2 (5-year NRFS: 99.1% vs. 97.4%, P = 0.38; 5-year OS: 93.8% vs. 88.0%, P = 0.14; Figure 1).

**Figure 2:**
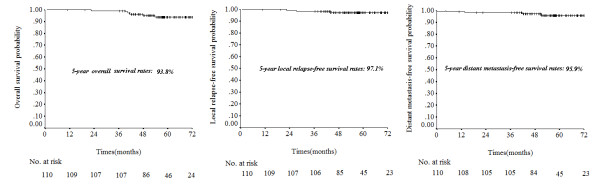
**Five year overall survival, local relapse-free survival and distant metastasis-free survival for patients with N0 disease according to the seventh edition of the American Joint Committee on Cancer (AJCC) nasopharyngeal carcinoma staging system**.

In this study, 110 patients (21.7%) were staged with N0 disease according to the seven edition of the AJCC staging system. In patients with N0 disease, the 5-year OS, LRFS, NRFS and DMFS rates were 93.8%, 97.1%, 99.1% and 95.9%, respectively (Figure 2). Of the N0 patients, five developed recurrence or metastasis; one of these developed local recurrence and distant metastases, one developed local and regional recurrence and then distant metastases, one developed isolated local recurrences and two developed isolated distant metastases.

### Local recurrence in N0 disease

Of the 110 N0 patients, three developed local recurrence; one of these patients had T2N0 disease and two patients had T4N0 disease. Age (≤50 years vs. > 50 years), gender, nasal cavity extension, oropharyngeal extension, parapharyngeal space extension, skull base erosion, paranasal sinus extension, hypopharyngeal extension, orbit extension, masticator space extension, cranial nerve palsy and intracranial extension were analyzed as prognostic factors in N0 disease. Univariate analysis revealed that paranasal sinus extension, orbit extension and intracranial extension were unfavorable prognostic factors for LRFS (all P < 0.01). Multivariate analysis was performed to test the significance of the independent variables, including all of the prognostic factors in the Cox proportional hazards model, and intracranial extension was shown to be a significant independent predictor of LRFS (HR: 29.5, CI: 2.6-334.4, P < 0.01).

### Distant metastasis and regional recurrence of N0 disease

Four of the 110 N0 patients developed distant metastasis; two of these patients had T1N0 disease, one had T2N0 disease and one had T4N0 disease. All of the prognostic factors were tested using univariate analysis and none were found to significantly determine DMFS.

Only one patient with T4N0 disease developed Level II lymph node recurrence, and this patient received prophylactic irradiation of the retropharyngeal area and the Level II, III, IV and V lymph node regions. At the time of lymph node recurrence, the patient was also found to have developed a local recurrence, and this patient developed distant metastases after 14 months.

### Survival of N0 disease according to AJCC T-classification

**Figure 3:**
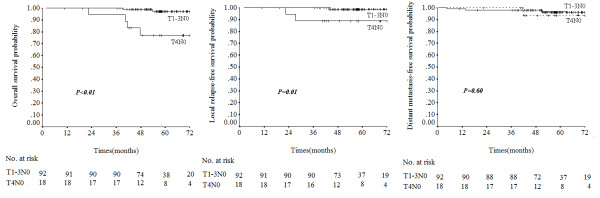
**Overall survival, local relapse-free survival and distant metastasis-free survival for different T-categories, according to the seventh edition of the AJCC nasopharyngeal carcinoma staging system**.

In T1N0, T2N0, T3N0 and T4N0 disease, the OS rates were 97.8%, 100%, 93.8% and 76.9%, respectively; and the DSS rates were same as the OS rates. The LRFS rates were 100%, 92.9%, 100% and 88.9% and the DMFS rates were 96.6%, 90.9%, 100% and 93.3%, respectively. The OS and DSS rates were higher in T1-3 N0 patients than T4N0 patients (P < 0.01 for both). Local failure rates were significantly higher in the T4N0 subset (P = 0.01), but there were no significant difference in DMFS between T1-3 N0 and T4N0 patients (P = 0.60, Figure 3). Of the 110 N0 patients, three had local failure; one of these had T2N0 disease and the other two had T4N0 with intracranial extension (Table 3).

**Table 3:** Five-year survival rates for N0 nasopharyngeal carcinoma patients according to different T-classifications of the seventh edition of AJCC staging system


**Stage**	**Death**		**Local recurrence**		**Distant metastasis**	
						
	**Event/*N***	**5-year OS (%)**	**Event/*N***	**5-year LRFS (%)**	**Event/*N***	**5-year DMFS (%)**

T1N0	1/60	97.8	0/60	100	2/60	96.6
T2N0	0/16	100	1/16	92.9	1/16	90.9
T3N0	1/16	93.8	0/16	100	0/16	100
T4N0	4/18	76.9	2/18	88.9	1/18	93.3

OS, overall survival; LRFS, local relapse-free survival; DMFS, distant metastasis-free survival

## Discussion

The 5-year relative survival rate for all NPC patients has increased from approximately 50% to 75% over the past ten years [15,18]. Advances in technology and the increasing availability of both diagnostic and therapeutic facilities have made significant contributions to this achievement. However, the introduction of new therapeutic interventions or technologies requires a reevaluation of the treatment outcomes and the patterns of failure in NPC patients with N0 disease after IMRT, in order to effectively guide treatment in the future.

### RLN metastasis classification

According to the sixth edition of the AJCC staging system, Tang et al. [13] and Tham et al. [14] proved that N0 NPC patients with RLN alone have a similar risk of distant metastasis to patients with N1 disease. The proposal that patients with RLN should be classified with N1 disease formed the basis of the revisions to the N0/N1 classifications in the seventh edition of the AJCC staging system. In this study, all patients underwent magnetic resonance imaging (MRI) examinations and received IMRT as their primary treatment. In patients with negative cervical lymph node involvement, the presence of RLN metastasis was found to negatively affect both DMFS, indicating it is still appropriate to classify RLN metastasis as N1 disease, even in the era of improved NPC treatment and diagnosis.

### Local recurrence

IMRT is considered to be an advantageous radiation treatment technique compared to conventional two-dimensional radiation. IMRT can deliver high-dose irradiation to defined tumor targets, while minimizing the dose delivered to the surrounding normal organs and tissues, thereby improving the therapeutic ratio of radiation therapy [7]. IMRT has improved the treatment outcome with respect to locoregional control, and in this study 5-year LRFS for all patients was 93.7%, similar to the local control rates of 90%-97% achieved in other studies using IMRT [8-11,19]. In N0 disease, we achieved an excellent local control rate of 96.3%.

### Distant metastasis

In NPC patients treated with two-dimensional conventional radiotherapy, prognostic factors such as parapharyngeal space extension, cranial nerve palsy and intracranial extension are validated prognostic factors for distant metastasis [5]. However, in our cohort of N0 patients treated with IMRT, no prognostic factor could significantly determine DMFS, which may be due to the excellent local control offered by IMRT, which reduces the rate of metastasis and may weaken the significance of potential prognostic factors.

### Regional recurrence

Radiation oncologists have previously treated all neck node levels in NPC comprehensively with definitive intent radiotherapy or prophylactic irradiation [20]. Several investigators confirmed that elective level II, III, and VA irradiation is suitable for NPC without lymph node metastasis [4,21]. Therefore, the volume of N0 disease that requires irradiation can vary among different doctors. As a result, only 43.6% N0 disease patients, who had no cervical lymph nodes or RLN metastasis based on MRI, received prophylactic irradiation to the upper neck lymph drainage region in this study. Only one failure was observed in a patient who developed Level II lymph node recurrence after receiving prophylactic irradiation of the retropharyngeal area and Level II, III, IV, and V lymph node regions. It should be emphasized that this patient developed a local recurrence, and so it is unclear whether the regions of lymph node recurrence received efferent lymphatics from the local recurrence

Using MRI, false negative diagnoses of metastases in the neck regions are relatively rare, with an occurrence rate of 0.5% [21]. Furthermore, high-dose irradiation of the neck area is associated with side effects, such as soft tissue fibrosis, which may adversely affect the patients' quality of life [22]. Our data confirmed that prophylactic irradiation which excludes the Level IV and the supraclavicular region does not increase the risk of regional recurrence in patients classified with N0 disease according to the seventh edition of the AJCC staging system using MRI again.

### Survival of N0 disease according to AJCC T-classification

T1N0, T2N0 and T3N0 showed excellent survival rates in this study. Tham et al. [11] reported excellent locoregional control in NPC, with no significant difference in the LRFS rates of T1, T2 and T3 patients. We focused on patients with N0 disease, in which the prognostic factor of lymph nodes metastasis was excluded, and found that the current TNM staging T-classification is becoming less powerful for segregation of patients into risk groups for local control and overall survival. In practice, locoregional control in T1-T3 patients should no longer be a major problem due to the improved outcomes after IMRT treatment, accurate geographic coverage of tumors assisted by CT-guided radiation treatment planning, increased diagnostic accuracy provided by MRI and PET and the intensive use of chemotherapy. Therefore, due to improved survival, it will become increasingly important to pay attention to the long-term complications of NPC treatment.

In this study, T4 patients had the poorest prognosis, and T4 disease was the most challenging to treat. Ng et al. also reported that advanced T4 disease remains difficult to treat [23]. Perhaps there are two main reasons. Firstly, intracranial extension is a significant independent predictor of LRFS in this study, as the adjacent brainstem and spinal cord cannot tolerate radiation doses at levels of 64 Gy in small volumes (1-10 mL) [24]. When IMRT is administered proximal to critical neurological structures, such as the brain stem, the very steep dose gradient contributes to an inadequate tumor dose and increases the risk of marginal failures. The risk of marginal failures may be reduced by increasing the precision of treatment using an adequate and very tight dose coverage of target volumes. Image-guided radiotherapy (IGRT) can potentially improve the accuracy of radiotherapy treatment delivery, particularly within high-dose gradients [25]. IMRT with IGRT could theoretically improve local control in T4N0 disease by reducing the incidence of marginal failures, and this hypothesis should be tested in the clinic. Secondly, another possible explanation is that larger tumors of T4 disease need higher radiation doses for tumor control due to the log-cell-kill principle of radiation treatment. This need could be solved by increasing the physical dose of radiation to an optimal level and/or by administering accelerated fractionation to the tumor. Whole-field simultaneous integrated-boost intensity-modulated radiotherapy with a dose > 70 Gy achieved excellent locoregional control, without an excess incidence of severe [26]. However, it must be emphasized that this was a retrospective study, and our conclusions need to be confirmed by future prospective studies.

## Conclusions

In summary, this study of NPC patients treated with IMRT at a single institution offers valuable information for the evaluation of prognosis in N0 disease in the modern era. The seventh edition of the AJCC N-staging system has improved the prognostic accuracy by upgrading RLN metastasis to N1 disease. Prophylactic irradiation excluding the Level IV and supraclavicular region does not increase the risk of regional recurrence in N0 disease. IMRT produces excellent survival rates in T1-3 N0 disease, therefore, it will be increasingly important to pay attention to the long-term complications of treatment in T1-3 N0 NPC patients. However, T4N0 disease remains a challenge with poorer survival rates, and additional improvements are required to achieve a favorable prognosis for these patients.

## Competing interests

The authors indicate no actual or potential conflicts of interest exist.

## Authors' contributions

The authors contributions are the following: Ying Sun (PhD, associate professor) and Ling-Long Tang (MD) contributed with literature research, study design, data collection, data analysis, interpretation of findings and writing of the manuscript. Lei Chen (MD), Wen-Fei Li (MD) and Yan-Ping Mao (MD) contributed with data collection. Li-Zhi Liu (MD, associate professor) and Li Li (PhD, professor) contributed with reviewing MR images. Ai-Hua Lin (PhD, professor) contributed with data analyses. Jun Ma (PhD, professor) contributed with data collection, study design, critical review of data analyses, interpretation of findings and critical edit of the manuscript. All authors read and approved the final manuscript.

## Pre-publication history

The pre-publication history for this paper can be accessed here:

http://www.biomedcentral.com/1471-2407/12/68/prepub
